# Predictive value of segmental extent of late gadolinium enhancement and peak circumferential systolic strain in predicting improvement and normalisation of dysfunctional segments post STEMI

**DOI:** 10.1186/1532-429X-17-S1-O11

**Published:** 2015-02-03

**Authors:** Jamal N Khan, John P Greenwood, Sheraz A Nazir, Miles Dalby, Nick Curzen, Simon Hetherington, Damian Kelly, Daniel J Blackman, Charles Peebles, Joyce Wong, Marcus Flather, Anthony Gershlick, Gerry P McCann

**Affiliations:** 1 Cardiovascular Sciences, University of Leicester, Leicester, UK; 2 University of Leeds, Leeds, UK; 3 Harefield Hospital, Middlesex, UK; 4 Southampton General Hospital, Southampton, UK; 5 Kettering General Hospital, Kettering, UK; 6 Royal Derby Hospital, Derby, UK; 7 University of East Anglia, Norwich, UK

## Background

Segmental infarct transmurality on late gadolinium enhancement (LGE) acutely post ST-elevation myocardial infarction (STEMI) has been shown to predict segmental function in a few small studies. The predictive power of segmental area of enhancement (n=45) and myocardial salvage (n=34) have been analysed in one small study each. Feature Tracking (FT) quantifies strain on routinely acquired SSFP cine images. There are no published large studies assessing segmental area of enhancement or FT-derived circumferential strain (Ecc) in predicting segmental function and improvement in the short and medium term post STEMI.

## Methods

CMR at 1.5T was performed in 164 patients (2624 myocardial segments) at 48hrs (baseline) and 9mth (follow-up) post STEMI. LV function was assessed using FT-derived Ecc and visual wall-motion scoring (WMS: 1=normal, 2=hypokinetic, 3=akinetic, 4=dyskinetic, 5=aneurysmal) on SSFP short-axis cine imaging. Segmental dysfunction was defined as WMS ≥2, improvement as a WMS decrease of ≥1, and normalisation where WMS=1 at follow-up. Segmental extent of LGE enhancement (SEE) was assessed on LGE short-axis imaging and graded on a 5-point scale: SEE-1: 0%, SEE-2: 1-25%, SEE-3: 26-50%, SEE-4: 51-75%, SEE-5: 76-100% of segmental area. Accuracy of baseline SEE and Ecc in predicting functional improvement and normalisation at follow-up was assessed using Receiver Operator Curves (ROC).

## Results

**Figure 1:**
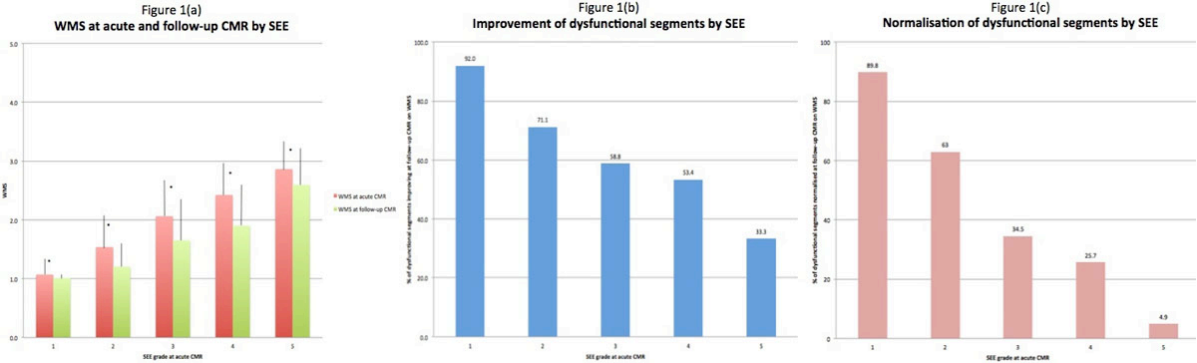
(a): WMS at baseline and follow-up CMR by SEE. (b): Improvement of dysfunctional segments by SEE. (c): Normalisation of dysfunctional segments by SEE.

32% of segments were dysfunctional at baseline and 19% at follow-up. With increasing SEE, segmental function worsened on WMS (Figure 1a). The proportion of dysfunctional segments improving (Figure 1b) or normalising (Figure 1c) decreased with increasing SEE. However 33% of dysfunctional SEE-5 segments improved at follow-up.

**Figure 2:**
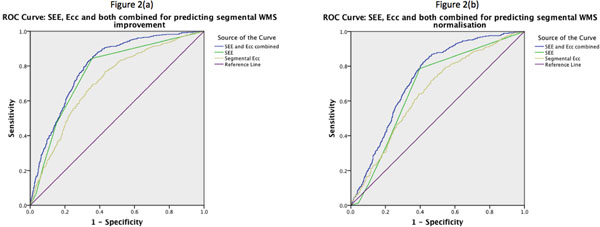
(a): ROC curves for accuracy of SEE, Ecc and both combined in predicting segmental functional (WMS) improvement in dysfunctional segments. (b): ROC curves for accuracy of SEE, Ecc and both combined in predicting segmental functional (WMS) normalisation in dysfunctional segments.

The odds-ratio (OR) for functional improvement decreased with increasing SEE (1: OR 23.1, 2: OR 4.9, 3: OR 2.9, 4: OR 2.3, p<0.001 vs. SEE-5). AUC for predicting improvement was 0.676. Segmental Ecc also predicted improvement (OR 1.04 per -1% Ecc), however with less accuracy (AUC 0.602, p=0.006 *vs*. SEE). Combining SEE and Ecc did not improve accuracy *vs*. SEE alone (AUC 0.687, p=0.67 *vs*. SEE, p=0.002 *vs*. Ecc) (Figure 2a).

The OR for functional normalisation decreased with increasing SEE (1: OR 170.3, -2: OR 33.1, 3: OR 10.2, 4: OR 6.7, p<0.001 vs. SEE-5). AUC for predicting normalisation was 0.775. Segmental Ecc also predicted normalisation (OR 1.06 per -1% Ecc), however with less accuracy (AUC 0.654, p<0.001 *vs*. SEE). Combining SEE and Ecc did not improve accuracy *vs*. SEE alone (AUC 0.796; p=0.36 *vs*. SEE, p<0.001 *vs*. Ecc) (Figure 2b).

## Conclusions

This is the largest study assessing the relationship between SEE, Ecc and functional improvement post STEMI. Baseline SEE strongly predicted segmental function post STEMI. SEE was a stronger predictor of improvement and normalisation in dysfunctional segments than baseline Ecc. Ecc provided no incremental value to SEE in predicting segmental improvement or normalisation. Functional improvement can occur even where SEE >75%.

## Funding

National Institute for Health Research (NIHR).

